# Melatonin ameliorates cuprizone‐induced reduction of hippocampal neurogenesis, brain‐derived neurotrophic factor, and phosphorylation of cyclic AMP response element‐binding protein in the mouse dentate gyrus

**DOI:** 10.1002/brb3.1388

**Published:** 2019-08-20

**Authors:** Woosuk Kim, Kyu Ri Hahn, Hyo Young Jung, Hyun Jung Kwon, Sung Min Nam, Jong Whi Kim, Joon Ha Park, Dae Young Yoo, Dae Won Kim, Moo‐Ho Won, Yeo Sung Yoon, In Koo Hwang

**Affiliations:** ^1^ Department of Anatomy and Cell Biology College of Veterinary Medicine, and Research Institute for Veterinary Science Seoul National University Seoul South Korea; ^2^ Department of Biochemistry and Molecular Biology College of Dentistry Research Institute of Oral Sciences Gangneung‐Wonju National University Gangneung South Korea; ^3^ Department of Anatomy College of Veterinary Medicine Konkuk University Seoul South Korea; ^4^ Department of Biomedical Science and Research Institute for Bioscience and Biotechnology Hallym University Chuncheon Korea; ^5^ Department of Anatomy College of Medicine Soonchunhyang University Cheonan South Korea; ^6^ Department of Neurobiology School of Medicine Kangwon National University Chuncheon Korea

**Keywords:** C57BL/6 mouse, cuprizone, hippocampus, melatonin, neurogenesis

## Abstract

**Introduction:**

The aim of this study was to investigate the effects of cuprizone on adult hippocampal neurogenesis in naïve mice. Additionally, we also studied how melatonin affects the neuronal degeneration induced by cuprizone.

**Methods:**

Eight‐week‐old male C57BL/6J mice were randomly divided into three groups: (a) the control group, (b) the group treated with cuprizone only, and (c) the group treated with both cuprizone and melatonin. Cuprizone was administered with food at 0.2% ad libitum for 6 weeks. Melatonin was also administered with tap water at 6 g/L ad libitum for 6 weeks; the animals were then euthanized for immunohistochemistry with Ki67, doublecortin (DCX), glucose transporter 3 (GLUT3), and phosphorylation of cyclic adenosine monophosphate (AMP) response element binding (pCREB); double immunofluorescence of neuronal nuclei (NeuN) and myelin basic protein (MBP); and Western blot analysis of brain‐derived neurotrophic factor (BDNF) expression to reveal the effects of cuprizone and melatonin on cell damage and hippocampal neurogenesis.

**Results:**

Administration of cuprizone significantly decreased the number of differentiating (DCX‐positive) neuroblasts and proliferating (Ki67‐positive) cells in the dentate gyrus. Moreover, cuprizone administration decreased glucose utilization (GLUT3‐positive cells) and cell transcription (pCREB‐positive cells and BDNF protein expression) in the dentate gyrus. Administration of melatonin ameliorated the cuprizone‐induced reduction of differentiating neuroblasts and proliferating cells, glucose utilization, and cell transcription.

**Conclusion:**

The results of the study suggest that cuprizone treatment disrupts hippocampal neurogenesis in the dentate gyrus by reducing BDNF levels and decreasing the phosphorylation of CREB. These effects were ameliorated by melatonin treatment.

## 1 INTRODUCTION

Copper is a trace element that plays crucial roles in many cellular processes. It is also a cofactor of enzymes and proteins associated with neural transmission and free radical scavenging (Rossi, Arciello, Capo, & Rotilio, 2006; Uriu‐Adams, Scherr, Lanoue, & Keen, 2010). Disturbance of copper metabolism results in neurological symptoms including mental retardation in humans as well as reduced myelination (Zimmerman, Matthieu, Quarles, Brady, & Hsu, 1976) and delayed development of the hippocampus in rats (Hunt & Idso, 1995). Cuprizone, a copper chelator, is widely used in the field of neuroscience because it induces demyelination when administered through food and this demyelination is reversible (Torkildsen, Brunborg, Myhr, & Bø, 2008; Zhen et al., 2017). Cuprizone disrupts cell metabolism and causes demyelination and eventually the death of oligodendrocytes and neurons in the brain and spinal cord (Gudi, Gingele, Skripuletz, & Stangel, 2014). The disruption of cell metabolism and demyelination may occur in pathological situations such as multiple sclerosis, which is a macerating neurological condition because it leads to immune‐mediated demyelination (Compston & Coles, 2008). In addition, a decrease in the activity of cytochrome oxidase and other mitochondrial enzymes, such as monoamine oxidase, in the brain occurs as a result of the administration of cuprizone (Venturini, 1973).

The hippocampus plays major roles in spatial memory and the consolidation of long‐term memory from short‐term memory (Goodman et al., 2010). Cells located in certain areas including the subgranular zone of the dentate gyrus can proliferate and differentiate into neuroblasts and granule cells throughout life. New neurons generated through hippocampal neurogenesis help in the acquisition of new skills and in movement coordination (Anacker & Hen, 2017; Opendak & Gould, 2015). The number of newly generated cells in the dentate gyrus decreases following the administration of several chemical toxins and during anxiety‐related disorders, while physical exercise and several anti‐anxiety drugs increase hippocampal neurogenesis (Ekdahl, Claasen, Bonde, Kokaia, & Lindvall, 2003; Kodama, Fujioka, & Duman, 2004; Tanaka et al., 2016; Yi et al., 2009; Yun et al., 2016). Ki67 is expressed in the nucleus during the active cell cycle, except during the resting (G_0_) and early G_1_ phases. Therefore, Ki67 is used as a marker for cell proliferation (Cooper‐Kuhn & Kuhn, 2002). DCX, which is a microtubule‐associated protein, is expressed in neuronal precursor cells, differentiating neuroblasts, and immature neurons, and thus, DCX is used as a marker for neuroblast differentiation (Karl et al., 2005). There is morphological evidence that cuprizone affects proliferating cells and neuroblasts in the rat dentate gyrus and progenitor cells in rat offspring (Abe, Tanaka, Kimura, Mizukami, Imatanaka, et al., 2015; Abe, Tanaka, Kimura, Mizukami, Saito, et al., 2015). Contrary to the aforementioned studies, it was reported in another study that myelin proteolipid protein‐null mice show a distinct proliferative response among progenitor cells in the subventricular zone without any changes in the number and proliferation of parenchymal oligodendrocyte progenitor cells (Gould et al., 2018). In addition, there is no study on the changes in microenvironmental conditions in the hippocampus after cuprizone treatment.

Melatonin (*N*‐acetyl‐5‐methoxytryptamine), a hormone produced by the pineal gland, is affected by the day and night cycle, and it regulates wakefulness (Hardeland, Pandi‐Perumal, & Cardinali, 2006). Melatonin has been proposed as a neuroprotective agent against neurodegenerative disease via direct and indirect antioxidant activity (Reiter, Manchester, & Tan, 2010). Melatonin has an antioxidative property and scavenges free radicals more effectively than do vitamins C and E (Korkmaz et al., 2009; Pieri, Marra, Moroni, Recchioni, & Marcheselli, 1994). Melatonin has positive effects about the hippocampus‐dependent cognitive function (Chen et al., 2017) as well as the hippocampal neurogenesis (Iggena, Winter, & Steiner, 2017; Yoo, Kim, Lee, et al., 2012; ). Additionally, it increases the number of proliferating cells and neuroblasts in the dentate gyrus of aged mice induced by D‐galactose treatment (Yoo, Kim, Lee, et al., 2012; ). Administration of melatonin dose‐dependently decreases cuprizone‐induced apoptosis by reducing caspase‐3, Bax, and heme oxygenase‐1 levels in the corpus callosum (Vakilzadeh et al., 2016). However, there is no study on the effects of melatonin on the hippocampus during cuprizone‐induced demyelination.

In the present study, we investigated the effects of cuprizone on the microenvironment of the hippocampus during adult neurogenesis in mice and the effects of melatonin on the hippocampus in a cuprizone‐induced demyelination model.

## 2 MATERIALS AND METHODS

### 2.1 Experimental animals

Eight‐week‐old male C57BL/6 mice were purchased from the Jackson Laboratory Co. Ltd. They were housed under standard conditions with feasible temperature (22 ± 2°C) and humidity (60 ± 5%) control and a 12:12‐hr light/dark cycle with ad libitum access to food and water. The handling and care of the animals conformed to the guidelines of current international laws and policies (National Institutes of Health [NIH] Guide for the Care and Use of Laboratory Animals, Publication No. 85–23, 1985, revised 1996) and were approved by the Institutional Animal Care and Use Committee of Seoul National University and Kangwon National University (KW‐171228‐2). All experiments were conducted with an effort to minimize the number of animals used and the physiological stress caused by the procedures employed in the present study. All experimental procedures were conducted according to ARRIVE guidelines (Kilkenny, Browne, Cuthill, Emerson, & Altman, 2010).

### 2.2 Experimental groups and treatment

The animals were divided into three groups (*n* = 15 in each group) as follows: (a) the normal diet‐fed (control) group, (b) the cuprizone‐containing diet‐fed (cuprizone) group, and (c) the melatonin‐supplied cuprizone (cuprizone + melatonin) group. Cuprizone diet was made by adding 0.2% cuprizone to chow diets. Melatonin was dissolved in water (6 mg/L) and administered ad libitum for 6 weeks.

### 2.3 Tissue processing

The animals (*n* = 9 in each group) were anesthetized with 2 g/kg urethane (Sigma‐Aldrich) 6 weeks after the first administration of cuprizone and melatonin. Subsequently, the thoracic cavity was opened and perfused transcardially with 0.1 M phosphate‐buffered saline (PBS, pH 7.4) followed by 4% paraformaldehyde in 0.1 M phosphate buffer (PB, pH 7.4) using a flexible tube (HV‐06409‐16, Masterflex) with needle as described in a previous study (Jung et al., 2019). The brains were then dissected and postfixed for 12 hr in the same fixative. The brain tissues were cryoprotected by overnight infiltration with 30% sucrose in 0.1 M PB. Serial brains were sectioned in the coronal plane at a thickness of 30 μm using a cryostat (Leica) and collected in six‐well plates containing PBS for further processing.

### 2.4 Immunohistochemistry for DCX, Ki67, GLUT3, and pCREB

The sections were processed under the same conditions to obtain comparable immunohistochemistry among groups as described in a previous study (Jung et al., 2019). Three sections from 1.82 to 2.32 mm posterior to the bregma according to a mouse atlas (Paxinos & Franklin, 2001), separated by intervals of 150 μm, were obtained from each animal. Each tissue section was sequentially treated with 0.3% H_2_O_2_ in PBS for 30 min and 10% normal goat serum in 0.1 M PBS for 30 min at 25°C. The sections were first incubated overnight with rabbit anti‐Ki67 (1:1,000; Abcam), rabbit anti‐doublecortin (DCX, 1:2,000, Abcam), rabbit anti‐glucose transporter 3 (GLUT3; 1:50, Santa Cruz Laboratory), or rabbit anti‐phosphorylated cAMP response element‐binding protein at Ser133 (pCREB, 1:400; Cell Signaling) at 25°C. The next day, the sections were treated with biotinylated goat anti‐rabbit IgG (1:200; Vector) for 2 hr at 25°C. Subsequently, the sections were treated with streptavidin–peroxidase complex (1:200; Vector) for 2 hr at 25°C. Thereafter, the brain sections were visualized by reaction with 3,3′‐diaminobenzidine tetrahydrochloride (DAB, Sigma) in 0.1 M Tris‐HCl buffer (pH 7.2) and mounted on gelatin‐coated slides. Sections were dehydrated with graded concentrations of alcohol and mounted in Canada balsam (Kanto Chemical).

### 2.5 Immunofluorescence staining for NeuN and MBP

To confirm localization of neuronal nuclei (NeuN) and myelin basic protein (MBP) in neurons, the mouse brain sections were processed using double immunofluorescence staining. Tissue sections from 1.82 and 2.32 mm posterior to the bregma according to a mouse atlas (Paxinos & Franklin, 2001), separated by intervals of 150 μm, were obtained from each animal. Each tissue section was sequentially treated with 10% normal goat serum in 0.1 M PBS for 30 min at 25°C. The sections were incubated overnight with a mixture of antisera of mouse anti‐NeuN (1:100; Merck Millipore) and rabbit anti‐MBP (1:200; Merck Millipore) at 25°C. The next day, after washing in PBS three times, the sections were treated with a mixture of both Cy3‐conjugated donkey anti‐rabbit IgG (1:500; Jackson ImmunoResearch) and Alexa Fluor 488 AffiniPure Donkey Anti‐Mouse IgG (1:500; Jackson ImmunoResearch) for 2 hr at 25°C. Thereafter, the sections were mounted on gelatin‐coated slides and in a water‐soluble mounting medium, Fluoromount‐G^®^ (SouthernBiotech).

### 2.6 Western blot analysis

To confirm the effects of cuprizone and/or melatonin on hippocampal neurogenesis, six mice in each group were sacrificed and used for Western blot analysis as described in a previous study (Yoo, Kim, Lee, et al., 2012). After removing the brain, the hippocampus was dissected with a surgical blade. The tissues were homogenized in 50 mM PBS (pH 7.4) containing 0.1 mM ethylene glycol‐bis(2‐aminoethylether)‐N,N,N′,N′‐tetraacetic acid (pH 8.0), 0.2% Nonidet P‐40, 10 mM ethylenediaminetetraacetic acid (pH 8.0), 15 mM sodium pyrophosphate, 100 mM β‐glycerophosphate, 50 mM NaF, 150 mM NaCl, 2 mM sodium orthovanadate, 1 mM phenylmethylsulfonyl fluoride, and 1 mM dithiothreitol (DTT). After centrifugation, protein levels were determined in the supernatants using a Micro BCA Protein Assay Kit with bovine serum albumin standards (Pierce Chemical). Samples containing 20 μg total protein were boiled in the loading buffer containing 150 mM Tris (pH 6.8), 3 mM DTT, 6% sodium dodecyl sulfate, 0.3% bromophenol blue, and 30% glycerol. The samples were then loaded onto polyacrylamide gel. After electrophoresis, the proteins were transferred to nitrocellulose transfer membranes (Pall Corp). To reduce background staining, the membranes were incubated in 5% nonfat dry milk in PBS containing 0.1% Tween‐20 for 45 min. The membrane was then incubated in rabbit anti‐brain‐derived neurotrophic factor (BDNF, 1:1,000, Proteintech). After incubation in the primary antibodies, the membranes were incubated in peroxidase‐conjugated anti‐rabbit IgG, and the signal was developed with a luminol‐based enhanced chemiluminescent kit (Pierce Chemical). The blot was scanned for the densitometric analysis of bands. The relative optical density (ROD) of each band was quantified using the Scion Image software (Scion Corp.). Data were normalized for β‐actin.

### 2.7 Data analysis

Analyses of the regions of the hippocampal dentate gyrus for DCX‐ and GLUT3‐positive cells were performed using an image analysis system and ImageJ software (NIH) as previously described (Jung et al., 2019; Yoo et al., 2016). Data were analyzed under the same conditions by two observers for each experiment in blinded conditions to avoid the bias. Digital images of the whole dentate gyrus were captured with a BX51 light microscope (Olympus) equipped with a digital camera (DP72, Olympus). Images were calibrated into an array of 512 × 512 pixels corresponding to a tissue area of 1,200 μm × 900 μm (100× primary magnification). Each pixel resolution had 256 gray levels, and the intensity of DCX and GLUT3 immunoreactivity was evaluated based on the ROD, which was obtained after transformation of the mean gray level using the following formula: ROD = log_10_ (256/mean gray level). The ROD of background staining was determined in unlabeled portions of the sections using Photoshop CC 2018 software (Adobe Systems Inc.), and this value was subtracted to correct for nonspecific staining using ImageJ software version 1.50. For MBP immunoreactivity, all hippocampal areas were selected and optical densities were measured using ImageJ software. Data are expressed as percentages of the control group (set at 100%).

The number of Ki67‐, pCREB‐, and NeuN‐positive cells in the dentate gyrus was counted using an analysis system with a computer‐based CCD camera (OPTIMAS software version 6.5; CyberMetrics^®^ Corporation; magnification, 100×). All counts from all sections were averaged.

### 2.8 Statistical analysis

Results are shown as mean ± standard error of mean (*SEM*). Statistical analysis of data was performed using one‐way analysis of variance (ANOVA), and further comparisons were assessed using Tukey's multiple‐range test in order to elucidate the effects of cuprizone and/or melatonin on hippocampal neurogenesis in mice. A *p*‐value <.05 was considered statistically significant.

## 3 RESULTS

### 3.1 Proliferating cells in the dentate gyrus

**Figure 1:**
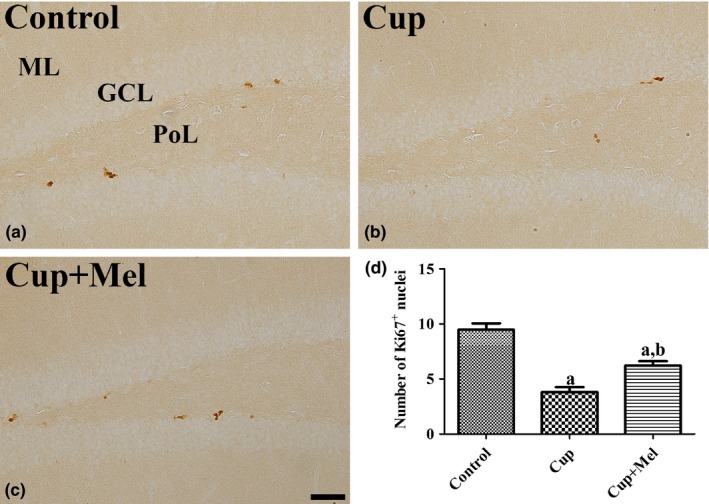
Immunohistochemistry for Ki67 in the hippocampal dentate gyrus of the control (a), cuprizone‐treated (Cup, b), and cuprizone + melatonin‐treated (Cup + Mel, c) groups. Note that there are few Ki67‐positive cells in the Cup group, while some Ki67‐positive cells are observed in mice in the control and Cup + Mel groups. GCL, granule cell layer; ML, molecular layer; PL, polymorphic layer. Scale bar = 50 μm. (d) Number of Ki67‐positive cells per section in all groups (*n* = 9 per group; ^a^ *p* < .05, significantly different from the control group; ^b^ *p* < .05, significantly different from the Cup group). The bars indicate the standard error of mean (*SEM*)

In the control group, Ki67‐positive proliferating cells were mainly observed in the subgranular zone of the dentate gyrus (Figure 1a). The number of Ki67‐positive cells per section was 9.5 in this group (Figure 1d). In the cuprizone group, the number of Ki67‐positive cells, which was 3.8, was significantly decreased compared to that in the control group (Figure 1b,d). In the cuprizone + melatonin group, the number of Ki67‐positive cells, which was 6.2, was significantly increased compared to that in the cuprizone group (Figure 1c,d).

### 3.2 Neuroblasts in the dentate gyrus

**Figure 2:**
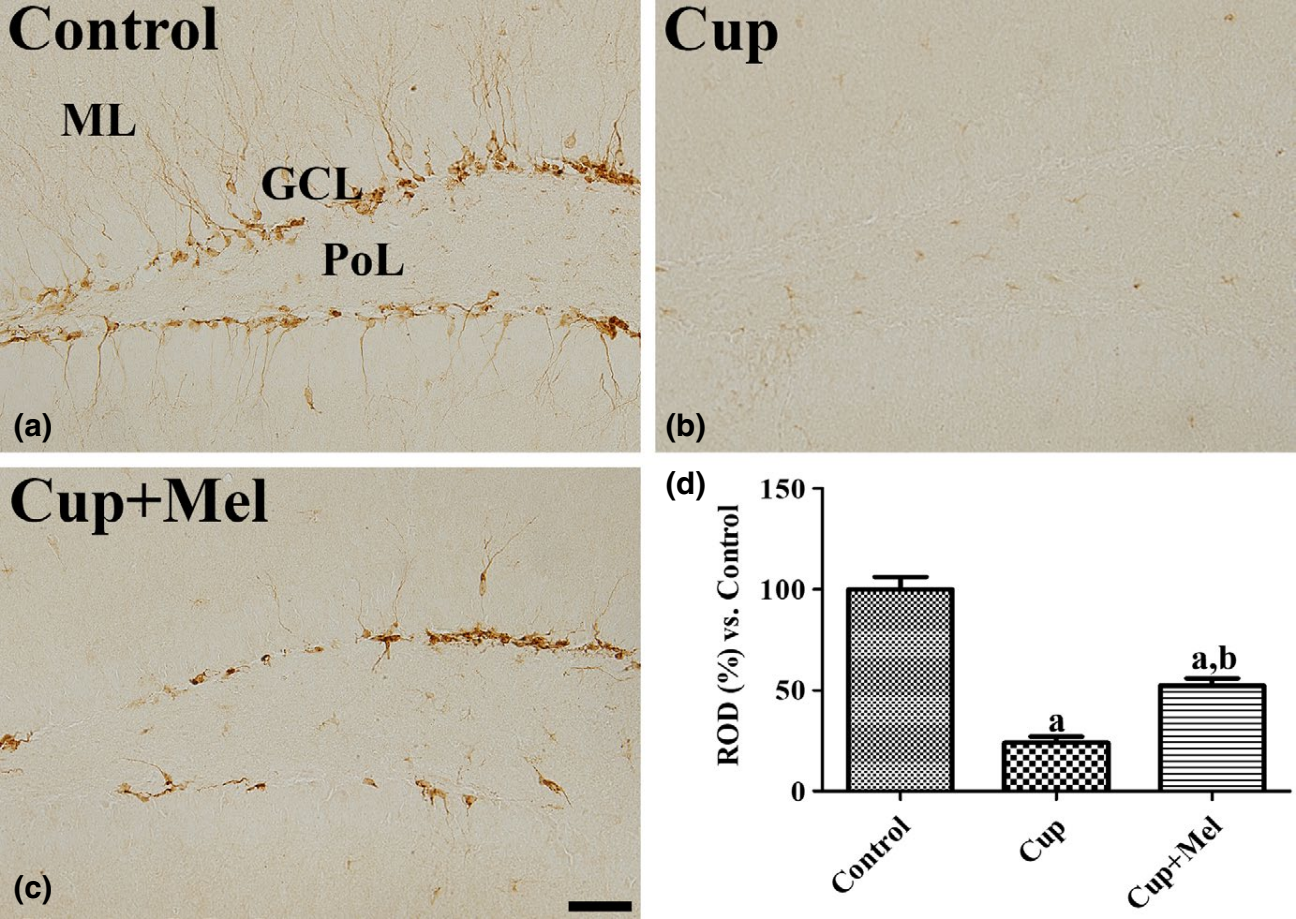
Immunohistochemistry for DCX in the hippocampal dentate gyrus of mice in the control (a), cuprizone‐treated (Cup, b), and cuprizone + melatonin‐treated (Cup + Mel, c) groups. DCX‐immunoreactive cell bodies and dendrites are detected in the subgranular zone and granule cell layer (GCL) of the dentate gyrus. Note that DCX‐immunoreactive cell bodies and their dendrites are scarcely rarely observed in these regions in the Cup group. GCL, granule cell layer; PoL, polymorphic layer. Scale bar = 50 μm. (d) The relative optical densities (RODs) expressed as a percentage of the value representing the DCX immunoreactivity in the dentate gyrus of mice in the control group are shown (*n* = 9 per group; ^a^ *p* < .05, significantly different from the control group; ^b^ *p* < .05, significantly different from the Cup group). The bars indicate the standard error of mean (*SEM*)

In the control group, DCX‐positive neuroblasts were observed in the subgranular zone of the dentate gyrus (Figure 2a). In the cuprizone group, the number of DCX‐positive neuroblasts was significantly decreased in the dentate gyrus compared to that in the control group (Figure 2b). In the cuprizone + melatonin group, the number of DCX‐positive cells in the DG was significantly increased compared to that in the cuprizone group (Figure 2c). The mean percentages of ROD were 23.9% in the cuprizone group and 52.3% in the cuprizone + melatonin group compared to 100% in the control group (Figure 2d).

### 3.3 Expression of GLUT3 in the hippocampal dentate gyrus

**Figure 3:**
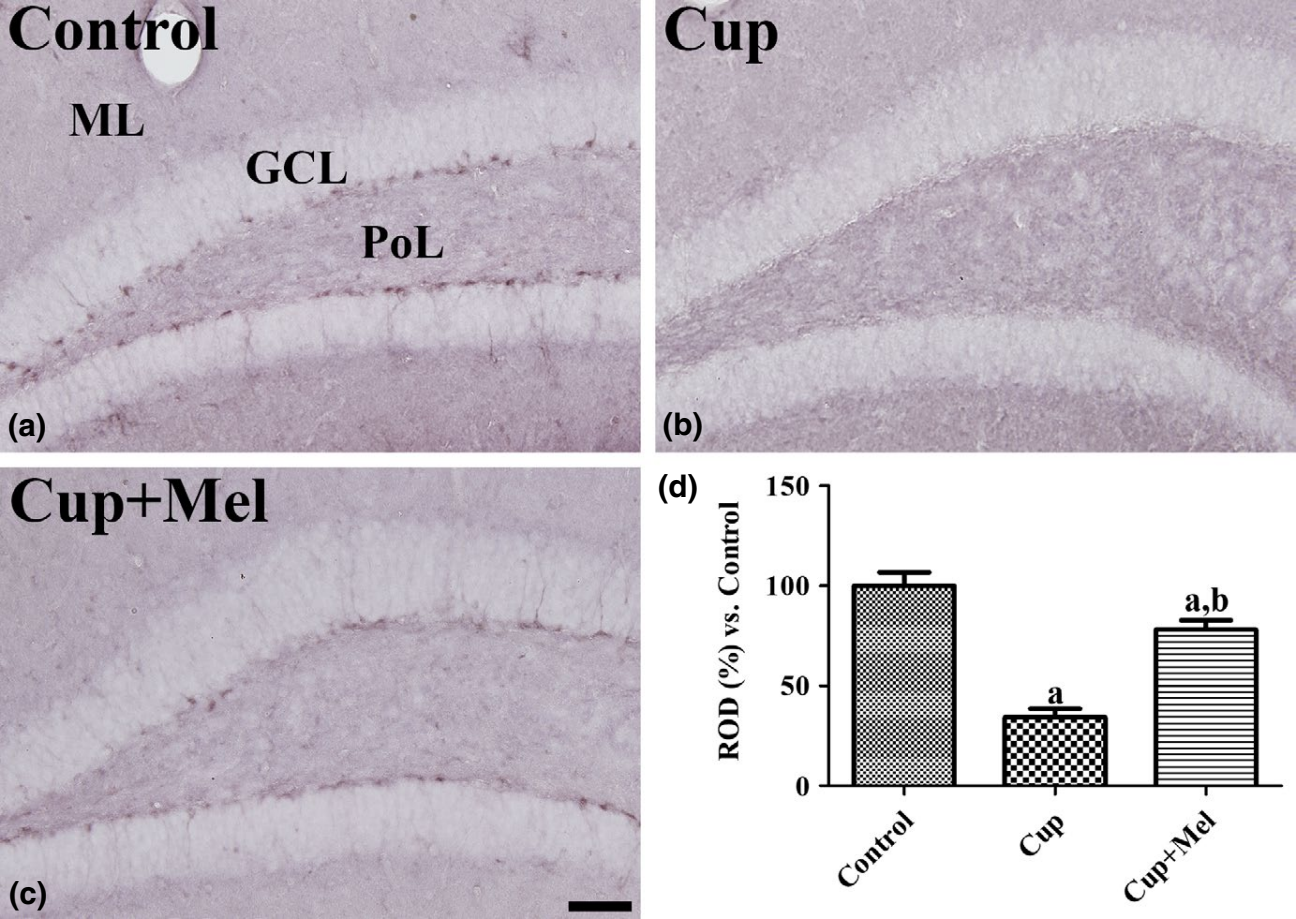
Immunohistochemistry for GLUT3 in the hippocampal dentate gyrus of mice in the control (a), cuprizone‐treated (Cup, b), and cuprizone + melatonin‐treated (Cup + Mel, c) groups. GLUT3‐immunoreactive structures are observed in the subgranular zone of the dentate gyrus. Note that GLUT3 immunoreactivity in the dentate gyrus of mice in the Cup group is lower than that in other groups. GCL, granule cell layer; ML, molecular layer; PoL, polymorphic layer. Scale bar = 50 μm. (d) The relative optical densities (RODs) expressed as a percentage of the value representing the GLUT3 immunoreactivity in the dentate gyrus of mice in the control group are shown (*n* = 9 per group; ^a^ *p* < .05, significantly different from the control group; ^b^ *p* < .05, significantly different from the Cup group). The bars indicate the standard error of mean (*SEM*)

Immunohistochemistry for GLUT3 was performed to evaluate glucose utilization in the hippocampal dentate gyrus after cuprizone and melatonin treatment. GLUT3‐immunopositive cells were mainly detected in the subgranular zone of the dentate gyrus in mice in the control group (Figure 3a). In the cuprizone group, GLUT3 immunoreactivity was significantly decreased in the dentate gyrus compared to that in the control group (Figure 3b). In the cuprizone + melatonin group, GLUT3 immunoreactivity was significantly increased compared to that in the cuprizone group (Figure 3c). The mean percentages of ROD were 34.3% in the dentate gyrus of the cuprizone group and 78.2% in the cuprizone + melatonin group compared to 100% in the control group (Figure 3d).

### 3.4 Expression of pCREB in the dentate gyrus

**Figure 4:**
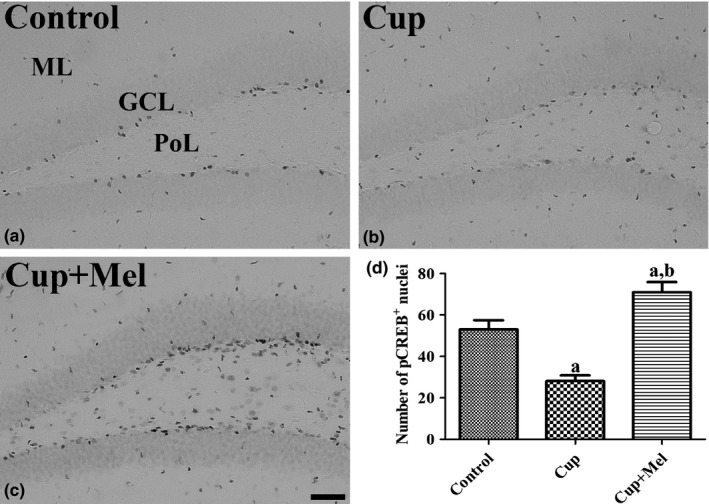
Immunohistochemistry for pCREB in the hippocampal dentate gyrus of mice in the control (a), cuprizone‐treated (Cup, b), and cuprizone + melatonin‐treated (Cup + Mel, c) groups. pCREB‐immunoreactive nuclei are mainly observed in the subgranular zone of the dentate gyrus. Note that few pCREB‐immunoreactive nuclei are observed in mice in the Cup group, but pCREB‐immunoreactive nuclei are abundant in mice in the Cup + Mel group. GCL, granule cell layer; ML, molecular layer; PL, polymorphic layer. Scale bar = 50 μm. (d) Number of pCREB‐positive cells per section in all groups (*n* = 9 per group; ^a^ *p* < .05, significantly different from the control group; ^b^ *p* < .05, significantly different from the Cup group). The bars indicate the standard error of mean (*SEM*)

In the control group, pCREB‐positive nuclei were mainly found in the subgranular zone of the dentate gyrus (Figure 4a). In the cuprizone group, the number of pCREB‐positive nuclei was significantly decreased in the dentate gyrus compared to that in the control group (Figure 4b). In the cuprizone + melatonin group, the number of pCREB‐positive nuclei was significantly increased in the subgranular zone of the dentate gyrus compared to that in the control and cuprizone groups (Figure 4c). The numbers of pCREB‐positive nuclei were 53.1, 28.1, and 71.0 in the control, cuprizone, and cuprizone + melatonin groups, respectively (Figure 4d).

### 3.5 Expression of NeuN in the dentate gyrus and MBP in the hippocampus

**Figure 5:**
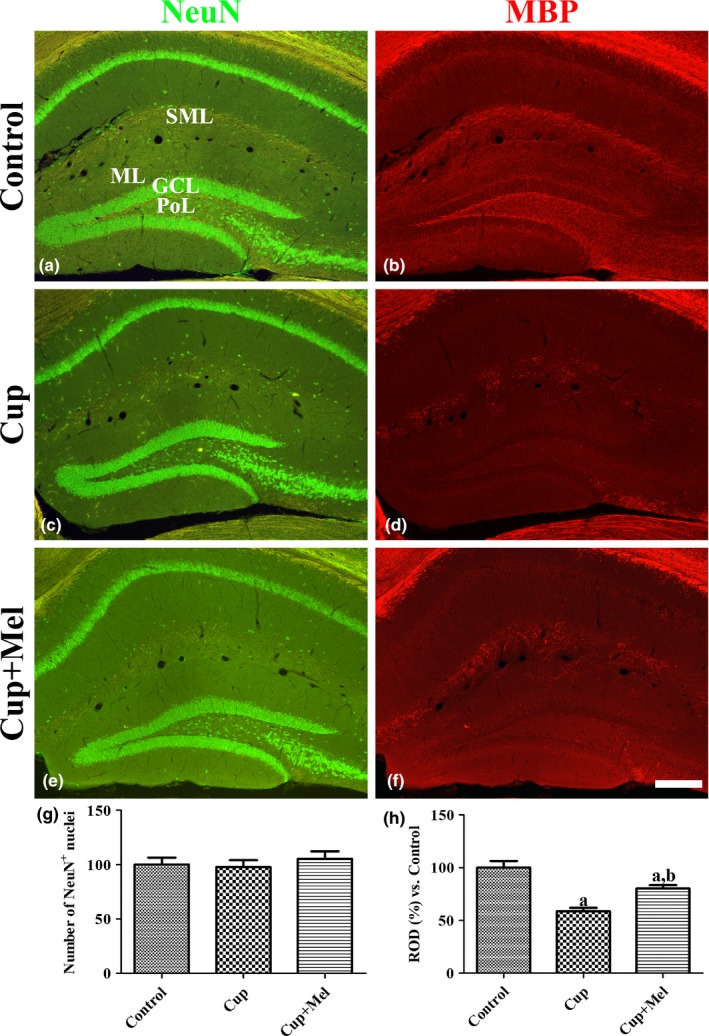
Immunofluorescence staining for NeuN (a, c, and e; green) and MBP (b, d, and f; red) in the hippocampus of mice in the control (a and b), cuprizone‐treated (Cup, c and d), and cuprizone + melatonin‐treated (Cup + Mel, e and f) groups. NeuN‐positive nuclei are found in all hippocampal subregions, and MBP‐immunoreactive structures are mainly observed in the alveus, stratum lacunosum‐moleculare (SML), molecular layer (ML), and polymorphic layer (PoL) of the dentate gyrus. Note that the number of NeuN‐positive nuclei in the dentate gyrus is similar in all groups, while MBP immunoreactivity is barely observed in the hippocampus of mice in the Cup group. GCL, granule cell layer. Scale bar = 200 μm. (g) Relative number of NeuN‐positive cells per section in the dentate gyrus of all groups and (h) the relative optical densities (RODs) expressed as a percentage of the value representing the MBP immunoreactivity in the hippocampus of mice in the control group are shown (*n* = 9 per group; ^a^ *p* < .05, significantly different from the control group; ^b^ *p* < .05, significantly different from the Cup group). The bars indicate the standard error of mean (*SEM*)

There were no differences in the number of NeuN‐positive mature neurons in the hippocampal dentate gyrus among the control, cuprizone, and cuprizone + melatonin groups (Figure 5a,c,e,g). However, there were significant differences in the MBP immunoreactivity among groups. In the control group, MBP immunoreactivity was mainly found in the hippocampal alveus, stratum lacunosum‐moleculare, molecular layer, and polymorphic layer of the dentate gyrus (Figure 5b). In the cuprizone group, overall MBP immunoreactivity was significantly decreased in the hippocampus compared to that in the control group (Figure 5d,h). In the cuprizone + melatonin group, MBP immunoreactivity increased in the hippocampus compared to that in the cuprizone group (Figure 5f,h).

### 3.6 BDNF protein expression in the hippocampus

**Figure 6:**
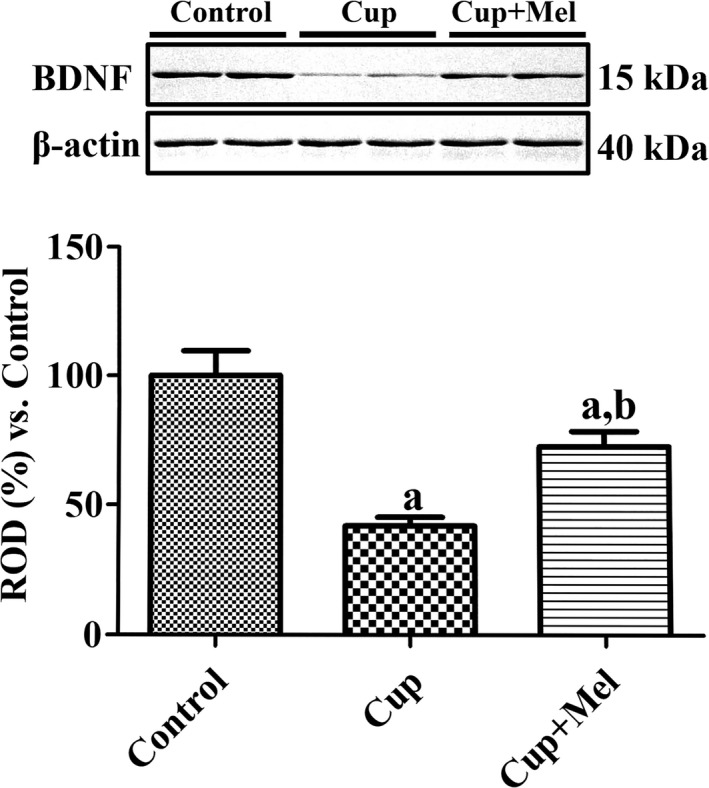
Western blot analysis of BDNF in the hippocampi of mice in the control, cuprizone‐treated, and cuprizone + melatonin‐treated groups. Values from western blot analysis are expressed as a ratio of the BDNF and β‐actin immunoblot bands in the control group (*n* = 6 per group; ^a^ *p* < .05, significantly different from the control group; ^b^ *p* < .05, significantly different from the Cup group). The bars indicate the standard error of mean (*SEM*)

Western blot analysis for BDNF was performed in all animal groups to confirm the cuprizone and melatonin treatment effect on BDNF expression in the hippocampus (Figure 6). In the cuprizone group, BDNF expression significantly decreased compared to that in the control group. The mean percentage of ROD was 41.8% compared to 100% in the control group (Figure 6). In the cuprizone + melatonin group, the expression level of BDNF significantly increased compared to that in the cuprizone group. The mean percentage of ROD was 72.5% compared to 100% in the control group (Figure 6).

## 4 DISCUSSION

Cuprizone, a copper chelator, caused neuronal degeneration including demyelination in various regions of the brain, but most studies focused on demyelination which occurred in the corpus callosum (Stidworthy, Genoud, Suter, Mantei, & Franklin, 2003). Cuprizone induces oxidative stress in the CNS, and cuprizone administration for several weeks results in neuronal degeneration such as CNS demyelination (Torkildsen et al., 2008; Zhen et al., 2017). In this study, we investigated the effects of melatonin on cuprizone‐induced demyelination in the hippocampal microenvironment during adult neurogenesis in mice by performing immunohistochemistry for Ki67, DCX, GLUT3, pCREB, and BDNF. To investigate the induction of demyelination by cuprizone, we conducted immunohistochemical staining for NeuN and MBP, which are expressed in mature neurons and myelin, respectively. Administration of cuprizone significantly decreased MBP immunoreactivity in the alveus, stratum lacunosum‐moleculare, and polymorphic layer of the dentate gyrus compared to that in the control group. However, the number of NeuN‐positive mature neurons was not significantly different between control and cuprizone‐treated mice. This result suggests that cuprizone causes demyelination in efferent fibers of pyramidal cells and afferent fibers such as perforant path and mossy fibers without significantly damaging neurons in the hippocampus. This result was consistent with that of a previous study reported that cuprizone intoxication induces demyelination in the stratum lacunosum‐moleculare and polymorphic layer of the dentate gyrus (Norkute et al., 2009). Additionally, there was no remarkable neuronal death in the hippocampus after cuprizone intoxication in adult animals (Praet, Guglielmetti, Berneman, Van der Linden, & Ponsaerts, 2014). In the present study, administration of melatonin significantly ameliorated the reduction in MBP‐immunoreactive structures in the alveus, stratum lacunosum‐moleculare, and polymorphic layer of the dentate gyrus. This result suggests that melatonin protects against demyelination due to cuprizone‐induced toxicity in the hippocampus and melatonin may be a possible therapeutic agent for the prevention of demyelination. It was reported that administration of melatonin dose‐dependently decreases cuprizone‐induced apoptosis‐related protein levels in the corpus callosum (Vakilzadeh et al., 2016).

In the present study, proliferating cells and neuroblasts in the hippocampal dentate gyrus were investigated using Ki67 and DCX immunostaining, respectively. Cuprizone administration significantly decreased the number of Ki67‐positive proliferating cells and DCX‐positive neuroblasts in the hippocampal dentate gyrus of mice. A general 28‐day toxicity study demonstrated that oral administration of 120 or 600 mg/kg cuprizone decreases the number of proliferating cell nuclear antigen‐positive proliferating cells and DCX‐immunoreactive neuroblasts in the rat dentate gyrus (Abe, Tanaka, Kimura, Mizukami, Saito, et al., 2015). Moreover, the addition of 0.4% cuprizone to the diet of dams caused a decrease in the number of progenitor cells in rat offspring (Abe, Tanaka, Kimura, Mizukami, Imatanaka, et al., 2015). However, Abe, Tanaka, Kimura, Mizukami, Imatanaka, et al. (2015) failed to demonstrate a significant decrease in MBP in the hippocampus, even though they observed some reduction in MBP mRNA levels. Melatonin administration affected not only the proliferation and differentiation of embryonic neural stem cells (Moriya, Horie, Mitome, & Shinohara, 2007) but also the stimulation of endogenous neurogenesis in an animal model of mild focal ischemia (Kilic et al., 2008). In the present study, the administration of melatonin to cuprizone group significantly increased the number of Ki67‐positive proliferating cells and DCX‐positive neuroblasts in the dentate gyrus compared to those in the cuprizone group. This result is consistent with that of a previous study which reported that melatonin ameliorates the reduced neurogenesis in the D‐galactose‐induced aging animal model (Yoo, Kim, Lee, et al., 2012). Our result is also supported by that of another study which showed that melatonin improves the reduced neurogenesis caused by irradiation (Manda, Ueno, & Anzai, 2009).

Glucose is transported into the brain by a family of facilitative transmembrane transport proteins, the GLUTs. GLUT3 plays a major role in glucose uptake in neurons (Rajakumar, Thamotharan, Menon, & Devaskar, 1998; Vannucci, Maher, & Simpson, 1997) because it facilitates glucose supply to the neurons even at low interstitial glucose concentrations. In the present study, we found that GLUT3 immunoreactivity was significantly decreased in the subgranular zone of the dentate gyrus after a 6‐week cuprizone diet compared to that after a normal diet. This means that cuprizone administration affects glucose transport and utilization in the proliferating cells or neuroblasts because GLUT3 immunoreactivity increases when neurogenesis is high, similar to the situation in newborn neurons (Jung et al., 2016) and in brain ischemia‐induced compensatory neurogenesis (Yoo et al., 2016). In the cuprizone + melatonin group, immunoreactivity of GLUT3 in the hippocampal dentate gyrus significantly increased compared to that in the cuprizone group. Melatonin administration increased the glucose consumption and utilization of neurons in the subgranular zone of the dentate gyrus. Therefore, melatonin administration ameliorated the reduced glucose transport and utilization in neurons as a result of cuprizone administration.

In the present study, we observed changes in the BDNF protein levels and pCREB immunoreactivity in the dentate gyrus after cuprizone toxicity and/or melatonin treatment because facilitation of CREB phosphorylation in primary hippocampal neurons was followed by acute and gradual stimulation of BDNF and increased neuronal plasticity (Ji et al., 2010). BDNF, a neurotrophin, promotes newborn neuron survival and maturation (Chan, Cordeira, Calderon, Iyer, & Rios, 2008; Choi, Li, Parada, & Sisodia, 2009), and the phosphorylation of CREB on Ser133 is a rate‐limiting step in the CREB‐signaling pathway (Gonzalez & Montminy, 1989). In the present study, we observed that the number of pCREB‐positive nuclei and BDNF expression decreased in the cuprizone group compared to that in the control group. This result suggests that cuprizone administration impairs hippocampal neurogenesis by decreasing pCREB protein and BDNF expression. In the cuprizone + melatonin group, the number of pCREB‐positive nuclei and the expression of BDNF in the hippocampal dentate gyrus significantly increased compared to that in the cuprizone group. Melatonin administration increased BDNF expression and phosphorylation of CREB protein in the hippocampus. This result is supported by our previous study which revealed that the administration of melatonin increases the number of pCREB‐positive nuclei in D‐galactose‐induced aging animal models (Yoo, Kim, Lee, et al., 2012). Phosphorylated CREB and BDNF are closely related, and this relationship contributes to neurogenesis (Begni, Riva, & Cattaneo, 2017). BDNF, secreted from the presynaptic neuron, binds to the tropomyosin receptor kinase B (TrkB) in the postsynaptic membrane. When BDNF binds to TrkB, several downstream signaling pathways, such as the mitogen‐activated protein kinase (MAPK), phospholipase C‐γ (PLC‐γ), and phosphoinositide‐3‐kinase (PI3K) pathways, are activated (Begni et al., 2017). When the MAPK signaling pathway is activated, extracellular signal‐regulated kinase 1/2 (ERK) is translocated into the nucleus, which then phosphorylates and activates transcription factors such as CREB (Grewal, York, & Stork, 1999; Shaywitz & Greenberg, 1999). Phosphorylated CREB binds to the BDNF promoter, which results in BDNF transcription (Shaywitz & Greenberg, 1999). This BDNF‐ERK‐CREB signaling plays a major role in neurogenesis by influencing neuronal cell survival, synaptic structure, and plasticity (Patapoutian & Reichardt, 2001). Therefore, melatonin treatment ameliorates the cuprizone‐induced reduction in hippocampal neurogenesis by upregulating BDNF expression and facilitating the phosphorylation of CREB in the hippocampus.

Collectively, cuprizone treatment significantly reduces the number of proliferating cells and neuroblasts in the hippocampus by reducing glucose utilization, BDNF expression, and the phosphorylation of CREB through demyelination, and not neuronal death. Administration of melatonin improves the demyelination in the hippocampus and the reduced cell proliferation and neuroblast differentiation by upregulating BDNF expression and facilitating glucose utilization and CREB phosphorylation. This result suggests that the cuprizone model can be used as an animal model of reduced hippocampal neurogenesis through demyelination, and not neuronal death.

## CONFLICT OF INTEREST

None declared.

## Data Availability

The data that support the findings of this study are available from the corresponding author upon reasonable request.
